# Zombie neurons in epilepsy: a burgeoning role for senescence in drug-resistant epilepsy

**DOI:** 10.1172/JCI189519

**Published:** 2025-03-03

**Authors:** Gemma L Carvill

**Affiliations:** Departments of Neurology, Pharmacology, and Pediatrics, Northwestern University Feinberg School of Medicine, Chicago, Illinois, USA.

## Abstract

Cellular senescence is a cell state induced by irreparable cellular damage. The hallmark of senescence is cell cycle exit, yet neurons, which are postmitotic from birth, have also been found to undergo senescence. Neuronal senescence is prevalent in aging as well as in neurodegenerative disease. However, a role for senescence in epilepsy is virtually unexplored. In this issue of the *JCI*, Ge and authors used resected brain tissue from individuals with drug-resistant epilepsy, a genetic knockout mouse model, and a chemoconvulsant mouse model, to demonstrate a subset of cortical pyramidal senescent neurons that likely contribute to the pathophysiology of epilepsy. These findings highlight senescence as a possible target in precision-therapy approaches for epilepsy and warrant further investigation.

## Cellular senescence in aging and neurodegenerative disease

**Figure 1:**
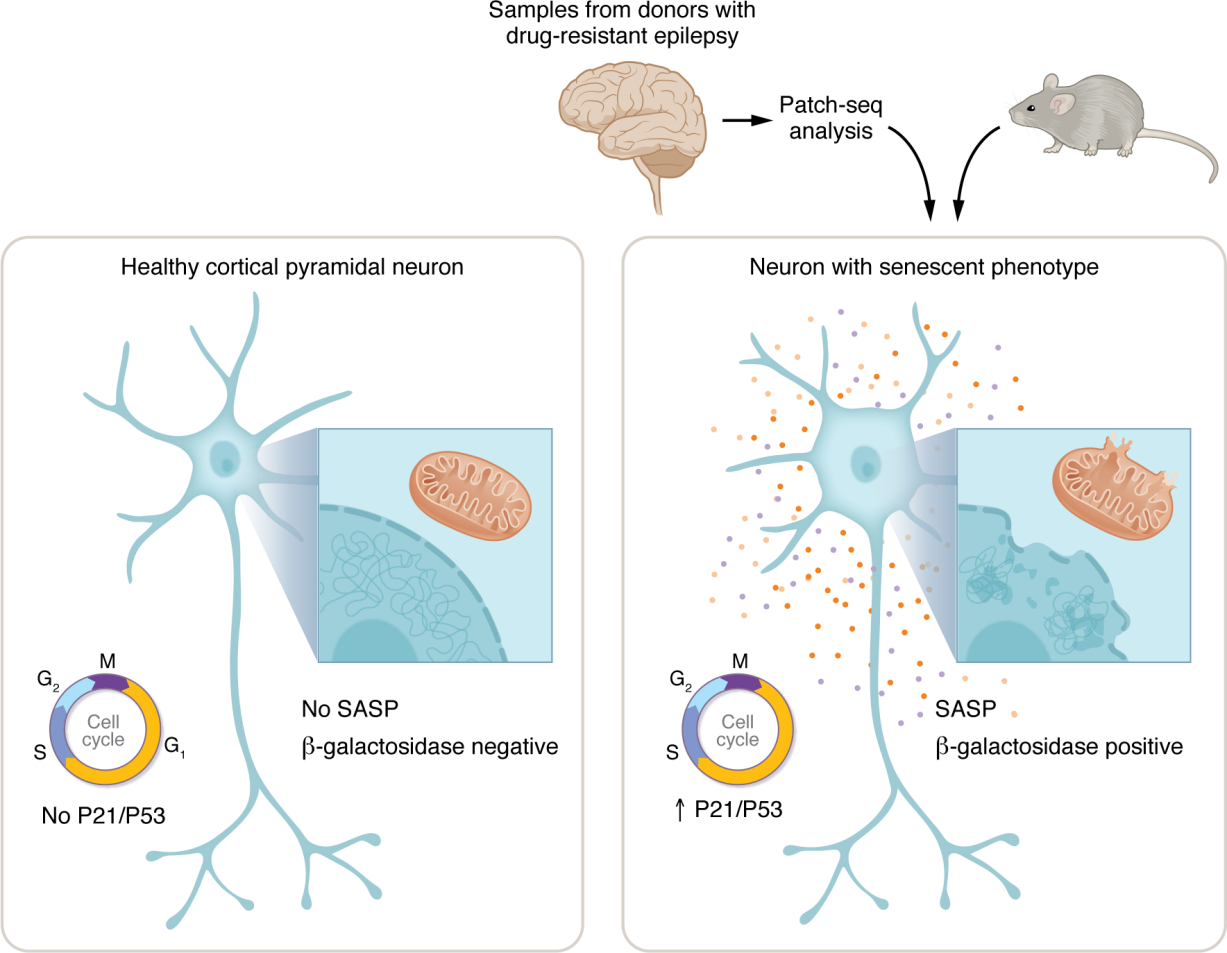
Senescent neurons contribute to drug-resistant epilepsy. Healthy neurons, which are postmitotic from birth, are typically paused in the G0 phase of the cell cycle. Senescent neurons feature a senescence-associated secretory-phenotype (SASP), consisting of inflammatory cytokines and chemokines. They also display DNA damage, decayed nuclear envelopes, and organelle damage, including mitochondria and lysosome degradation. Ge et al. performed patch-seq on neurons from individuals with drug-resistant epilepsy, characterizing neuronal morphological, electrophysiological, and transcriptional profiles. This strategy enriched a subpopulation of pathological cortical pyramidal neurons possessing a senescent phenotype. Neurons with this phenotype expressed markers of cell senescence, including β-galactosidase. They also showed increased expression of cell cycle inhibitors (e.g. P21, P53) typically associated with cell cycle arrest. Notably, a mouse model of chronic epilepsy also induced senescent neurons.

Cellular senescence is a cell state somewhere between life and death. Generally, when cells are irreparably damaged, programmed cell death occurs via apoptosis. However, under certain conditions, rather than cell death, cells can enter senescence, an apoptotic-resistant state associated with mitotic arrest. While the hallmark of senescence is exit of the cell cycle, neurons, which are postmitotic from birth, can also undergo senescence. This cell state in neurons is characterized by increased expression of cell cycle inhibitors (e.g., P21, P53), DNA damage, altered autophagy, epigenetic erosion (e.g., breakdown of the lamin nuclear envelope), and organelle dysfunction (especially mitochondria and lysosomes). In addition, the senescence-associated secretory-phenotype (SASP), consisting of inflammatory cytokines and chemokines, can occur (Figure 1).

Neuronal senescence is a well-established phenomenon in aging that can be induced by DNA damage, ROS, or oncogenic signaling. As such, neuronal senescence is increasingly implicated in neurodegenerative disease, including Alzheimer’s disease (1, 2). However, senescence has not been widely described nor studied in epilepsy. A single, hypothesis-driven study was recently published by Ribierre and colleagues demonstrating a cellular senescence signature in resected epilepsy tissue from individuals with focal cortical dysplasia type II (FCDII), as well two mTORopathy mouse models (*Depdc5* and *Mtor*) (3). In this issue of the *JCI*, Ge et al. performed unbiased single-cell resolution profiling of epileptic tissue from individuals with drug refractory epilepsy to reveal a common senescence phenotype in affected neurons, corroborating via distinct methodologies and models a role for senescence in epilepsy (4).

## Dissecting cellular heterogeneity in the epilepsies

One of the major challenges in dissecting the pathogenic mechanisms in epilepsy is that not all neurons participate in seizure generation and propagation, rather, specific neurons or networks are implicated. Thus, single cell resolution technologies are increasingly being implemented in the heterogenous epilepsies to probe disease mechanisms. For example, combinatorial transcriptome and protein expression single-cell profiling of resected epilepsy tissue from individuals with drug-resistant epilepsy revealed activation of microglia and infiltration of proinflammatory immune cells, consistent with a proinflammatory response and supporting an immune microenvironment in epilepsy (5).

One particularly attractive approach for studying the brain at single-cell resolution is patch-seq, which allows the simultaneous profiling of the electrophysiological, molecular, and morphological properties of a single neuron. This is achieved by traditional whole-cell electrophysiological patch-clamp recordings followed by aspiration of the cellular contents and single-cell RNA-seq, and, finally, morphological characterization using biocytin filling (6). This approach has been implemented to create robust atlases of neuronal subtypes in the human, primate, and mouse brain (7, 8) and is an attractive technology for studying epilepsy where simultaneous electrophysiological and molecular properties of neurons related to epilepsy can be studied. Ge et al. performed patch-seq in 197 neurons from 36 individuals living with drug-resistant epilepsy to reveal a common senescence phenotype in neurons (4).

## Senescence and epilepsy

The epilepsies are a heterogenous group of disorders characterized by hypersynchronous activity in the brain that manifests clinically as seizures. The highest unmet clinical need is for those individuals living with drug-resistant epilepsy, where antiseizure medications, as well as surgical and stimulation-based interventions, are ineffective, and ongoing seizure activity severely limits quality of life. The drug-resistant epilepsies comprise multiple subtypes, including the FCDs and temporal lobe epilepsies (TLE). FCD is usually captured on MRI and features balloon cells in the white matter and dysmorphic neurons in the grey matter, while TLE is characterized by hippocampal sclerosis and neuronal loss. Given the seemingly disparate pathologies, a unifying biological pathway to target and treat the drug-resistant epilepsies is highly desirable.

Ge et al. analyzed patch-seq data from 197 neurons collected from 36 individual donor samples from individuals with drug-resistant epilepsy with FCD and TLE. The electrophysiological properties and transcriptomes of these neurons were leveraged to identify a subset of cortical pyramidal neurons that had increased gene expression of senescence markers including cyclin inhibitors (*CDKN1A* [encoding P21], *CCL2,* and *NFKBIA*) and SASP-related interleukins and chemokines. Via multiple imaging studies (RNA and protein), Ge and colleagues validated this senescence phenotype in resected tissue from drug resistant epilepsy samples with different pathologies by increased abundance of cell-cycle inhibitors (P21, P53), the DNA-damage marker H2AX, and the senescence marker b-galactosidase (SA-β-Gal), as well as reduction of the nuclear integrity marker, Lamin B1. These phenotypes were specific to neurons with increased neurofilament and larger soma that are characteristic of pathology-related neurons in FCD. Finally, in addition to these experiments in human resected tissue, both a genetic neuronal knockout mTORopathy (*Pten*) and a chemoconvulsant mouse model of epilepsy were used to demonstrate increased senescence markers in chronic epilepsies, but not in acute seizures (Figure 1) (4).

## Clinical implications and unanswered questions

Collectively, the recent publications by the Ribierre research group and the Ge lab reveal a senescence phenotype in resected tissue from individuals with drug-resistant epilepsy from a wide spectrum of pathologies (FCD, TLE, and tumor-related epilepsy) (3, 4). Moreover, both teams validate these findings in genetic (*Depdc5/Pten/mtor*) and chemoconvulsant mouse models. These independent studies also garnered support for the ‘senescence theory’ via different methodologies, adding further weight to their conclusions. In particular, the Ribierre group proposed a senescence theory based on the known relationship between the mTOR pathway and a 15 year old study showing balloon cell cycle exit (3, 9). Ge et al. however, used an unbiased single-cell combinatorial electrophysiological and transcriptomic approach (4). Yet, both groups coalesced on the same conclusions and support for the role of senescence in drug resistant epilepsy (3, 4).

Overall, these studies suggest that senescence is indeed a characteristic of the drug-resistant epilepsies of diverse pathology. However, there were some discrepancies that warrant further study. First, while Ribierre et al. identified epileptiform activity in resected tissue using multielectrode arrays, Ge et al. observed no electrophysiological changes using patch clamp in pathological neurons (3, 4). Thus, further work is needed to dissect cell-autonomous and noncell-autonomous properties of these senescent neurons and the impact on a network disorder. Despite these unanswered questions, these studies highlight senescence as a potential pathology-targeting precision therapy. Indeed, the use of the senolytic drugs (dasatinib/quercetin) decreased the number of senescent cells and reduced seizure frequency in the mTOR mouse model (3), motivating further study. Another attractive strategy is the use of senomorphic agents, which can reverse a senescent phenotype rather than destroying senescent neurons, which are irreplaceable. Future preclinical and clinical trials should be pursued leveraging the senotherapies as a potential treatment for drug-resistant epilepsies.

## Version 1 03/03/2025

Electronic publication
